# Development and validation of a nomogram model for predicting venous thromboembolism risk in lung cancer patients treated with immune checkpoint inhibitors: A cohort study in China

**DOI:** 10.1002/cam4.70115

**Published:** 2024-08-20

**Authors:** Guanzhong Liang, Zuhai Hu, Qianjie Xu, Guixue Wang, Ying Wang, Xiaosheng Li, Wei Zhang, Haike Lei

**Affiliations:** ^1^ Chongqing Cancer Multi‐omics Big Data Application Engineering Research Center Chongqing University Cancer Hospital Chongqing China; ^2^ Department of Health Statistics, School of Public Health Chongqing Medical University Chongqing China; ^3^ MOE Key Lab for Biorheological Science and Technology, State and Local Joint Engineering Laboratory for Vascular Implants College of Bioengineering Chongqing University Chongqing China; ^4^ Chongqing Key Laboratory of Translational Research for Cancer Metastasis and Individualized Treatment Chongqing University Cancer Hospital Chongqing China

**Keywords:** immune checkpoint inhibitors, lung cancer, nomogram, venous thromboembolism

## Abstract

**Objective:**

Venous thromboembolism (VTE) poses a significant threat to lung cancer patients, particularly those receiving treatment with immune checkpoint inhibitors (ICIs). We aimed to develop and validate a nomogram model for predicting the occurrence of VTE in lung cancer patients undergoing ICI therapy.

**Methods:**

The data for this retrospective cohort study was collected from cancer patients admitted to Chongqing University Cancer Hospital for ICI treatment between 2019 and 2022. The research data is divided into training and validation sets using a 7:3 ratio. Univariate and multivariate analyses were employed to identify risk factors for VTE. Based on these analyses, along with clinical expertise, a nomogram model was crafted. The model's predictive accuracy was assessed through receiver operating characteristic (ROC) curves, calibration curves, decision curve analysis, clinical impact curve, and other relevant metrics.

**Results:**

The initial univariate analysis pinpointed 13 potential risk factors for VTE. The subsequent stepwise multivariate regression analysis identified age, Karnofsky performance status, chemotherapy, targeted, platelet count, lactate dehydrogenase, monoamine oxidase, D‐dimer, fibrinogen, and white blood cell count as significant predictors of VTE. These 10 variables were the foundation for a predictive model, illustrated by a clear and intuitive nomogram. The model's discriminative ability was demonstrated by the ROC curve, which showed an area under the curve of 0.815 (95% CI 0.772–0.858) for the training set, and 0.753 (95% CI 0.672–0.835) for the validation set. The model's accuracy was further supported by Brier scores of 0.068 and 0.080 for the training and validation sets, respectively, indicating a strong correlation with actual outcomes.

**Conclusion:**

We have successfully established and validated a nomogram model for predicting VTE risk in lung cancer patients treated with ICIs.

## 1 INTRODUCTION

Venous thromboembolism (VTE) refers to the abnormal formation of blood clots within the venous system, which can lead to the obstruction of blood vessels, including deep vein thrombosis (DVT) and its complications, as well as pulmonary embolism (PE).VTE is the second leading cause of death in cancer patients. The incidence of VTE associated with immune checkpoint inhibitors (ICIs) varies across studies. In a multicenter retrospective cohort study,^1^ it was found that the cumulative incidence of venous thromboembolism in patients with non‐small cell lung cancer (NSCLC) after treatment with ICIs was 14.8%. Another study involving Chinese lung cancer patients treated with ICIs reported a VTE incidence rate of 9.4%.^2^ A study has found that cancer patients suffering from VTE have a one‐year survival rate of 12% compared to 36% for those without VTE.^3^ Lung cancer ranks among the cancer types with the highest risk of developing VTE, where its prevalence among lung cancer patients is estimated to be between 2% and 15%. Notably, lung cancer patients who experience VTE exhibit a mortality rate approximately 50% higher than their counterparts without VTE.^4^, ^5^

Lung cancer remains a leading cause of cancer‐related mortality worldwide, exhibiting exceptionally high incidence and mortality rates in China.^6^ The introduction of ICIs, including anti‐Programmed Death Receptor‐1/Programmed Death Receptor‐Ligand 1(anti‐PD‐1/PD‐L1) and Cytotoxic T lymphocyte associate protein‐4(CTLA‐4) therapies, has markedly improved lung cancer patient outcomes. Nonetheless, the treatment is not without its challenges. Specifically, immune‐related adverse events triggered by these therapies, especially those involving systemic inflammation and impacting the hematological system, can increase the risk of VTE. VTE's occurrence as an immune‐related adverse event in the context of cancer immunotherapy, potentially driven by interleukin‐8 and myelosuppressive cell pathways, remains an area of ongoing research.^7^ Emerging evidence, particularly from two retrospective studies, highlights an elevated incidence of VTE among patients treated with ICIs, noting an uptick following the onset of therapy.^8^, ^9^ The risk of VTE in cancer patients receiving ICI treatment is comparable to that associated with cytotoxic chemotherapy.^10^ Furthermore, combined treatment regimens involving ICIs are associated with a markedly higher cumulative incidence of VTE compared to ICI monotherapy.^11^ Even as the awareness of increased VTE risk among cancer patients treated with ICIs grows, the specific risk factors and the applicability of established risk assessment tools, such as the Khorana score, are still under scrutiny.^12^ Given the scarcity of data on ICI‐related VTE in lung cancer and the limitations of existing risk assessment models like the Caprini and Khorana scales, our research utilized a nomogram model. This model aims to precisely forecast the high‐risk group for VTE among lung cancer patients post‐ICI therapy, aiming to bolster the diagnostic precision for VTE detection.

The nomogram model, grounded in multivariate regression analysis, serves as a predictive framework by merging multiple prognostic factors and illustrating their interrelationships through proportionally scaled line segments on a unified plane. This facilitates the calculation of individual outcome event probabilities, employing a graphical method of risk assessment that has been explored across various solid tumors.^13^, ^14^ Despite its proven utility, there currently lacks specific data for predicting VTE risk in lung cancer patients undergoing ICI therapy. In response to this gap, we employed the nomogram model to assess the risk of VTE among lung cancer patients treated with ICIs, considering a range of factors, including age and gender, among others. Our objective is to pinpoint individuals at heightened risk, offering a valuable tool for proactive VTE screening, prevention, and management within this patient cohort.

### 1.1 Research design and population

**FIGURE 1:**
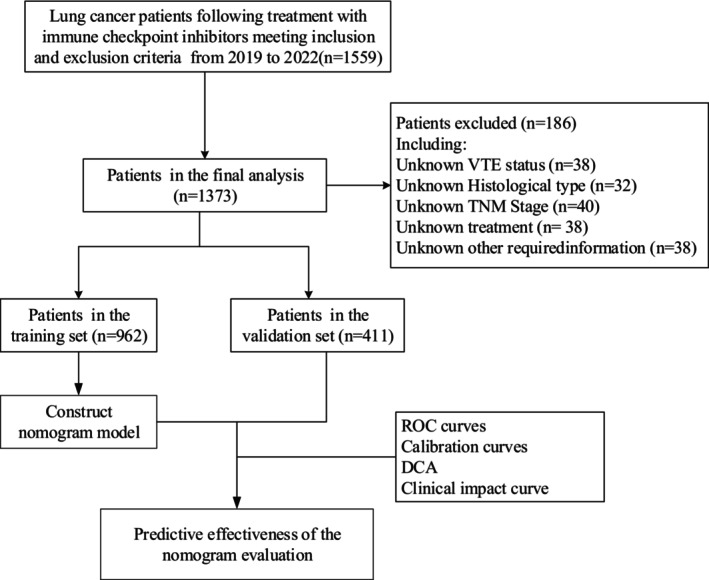
Detailed chart of inclusion and exclusion in the study.

The data for this study were gathered from lung cancer patients undergoing ICI inpatient treatment at Chongqing University Cancer Hospital between 2019 and 2022 (Figure 1). Each patient received a confirmed lung cancer diagnosis through tissue or cell pathology analysis. Lung cancer staging followed the guidelines outlined in the eighth edition of the UICC TNM standard by the Union for International Cancer Control. All Non‐squamous NSCLC patients included in the study were negative for mutations in genes associated with sensitivity, including EGFR, ALK, ROS‐1and BRAF. Exclusion criteria comprised adolescents (<18 years), individuals with a prior history of VTE, those currently under anticoagulation therapy or with a history of such treatment, individuals with concurrent infections, autoimmune diseases, other coexisting tumors, and patients with incomplete data. The dataset was partitioned into training and validation sets using a 7:3 ratio. The study was conducted following the guidelines of the Helsinki Declaration and obtained approval from the Ethics Committee of Chongqing University Cancer Hospital (Approval Number: CZLS2023343‐A).

### 1.2 Data collection and definition

Data were exclusively sourced from Chongqing University Cancer Hospital in China, encompassing comprehensive information from lung cancer patients who underwent ICI therapy. The treatments collected in the study were the primary treatments received by the patients after they were diagnosed with lung cancer. This dataset includes detailed patient demographics such as age, gender, Body Mass Index (BMI), Karnofsky Performance Status (KPS) score, TNM staging, surgical history, radiotherapy history, chemotherapy history, targeted therapy, platelet count (PLT), lactate dehydrogenase (LDH), monoamine oxidase (MAO), prothrombin time (PT), activated partial thromboplastin time (APTT), D‐dimer, fibrinogen (FIB), fibrin degradation products (FDP), T cell, B cell, NK cell ratios, white blood cell (WBC), and hemoglobin (Hb), making up a total of 22 indicators. In the article's modeling, the data for patients who did not experience VTE were extracted from the results obtained during their ICI treatment. For patients who experienced VTE, their blood parameter data was collected at the time of the VTE diagnosis. All VTE patients are diagnosed by clinical specialist physicians in conjunction with ultrasound or imaging examinations.

### 1.3 Statistical analysis

We randomly partitioned all patients into training and validation sets using R software's “caret” package, with a fixed random seed in a 7:3 ratio. For normally distributed data, Mean ± SD was used for description, and the *t*‐test was used for comparison. For non‐normally distributed data, the median (M) and interquartile range (IQR) were used for description, and non‐parametric tests were used for comparison. The analysis started with a univariate Logit model, with initial tests for all variables, followed by multivariable logistic stepwise regression for variables with *p* < 0.2 to selectively identify significant feature variables. These selected features from the stepwise regression were employed to construct the nomogram model. The model's accuracy and practicality were evaluated using ROC curves, calibration curves, decision curve analysis (DCA), and clinical impact curves (CIC). The Hosmer‐Lemeshow statistic measures the inconsistency between the probabilities predicted by a model and the actual observed outcomes. A lower statistic value denotes better model calibration, reflecting a closer match between the model's predictions and the observed realities. All statistical analyses were conducted using R software (version 4.3.1, https://www.R‐project.org), with a two‐sided *p* < 0.05 deemed statistically significant.

## 2 RESULTS

### 2.1 Demographic baseline characteristics

Between 2019 and 2022, 1373 lung cancer patients participated in this study, of which 123 patients experienced VTE, with an average VTE time of 5.86 months. The median follow‐up time is 12.9 months. Table 1 presents the patients' basic information, clinical diagnoses, treatment details, and their correlation with VTE. Among all VTE patients, 109 patients were DVT patients (88.62%), 8 patients were PE patients (6.50%), and 5 patients were DVT + PE patients (4.07%). The average age was 59.75 ± 9.25 years. Of the total, 258 individuals were female (18.79%), while 1115 were male (81.21%). Tumor TNM staging showed 1137 cases (82.81%) at stage IV. Among the patients, 599 (43.63%) underwent surgery, while 563 (41.01%) underwent radiotherapy. Chemotherapy was administered to 1294 cases (94.25%), and targeted therapy was given to 550 cases (40.06%). The average KPS score was 78.10 ± 9.39, with an average WBC count of 8.72 ± 4.52, average PLT count of 230.00 ± 84.95, average LDH level of 232.16 ± 125.18, average D‐dimer level of 1.29 ± 1.90, and average FIB level of 4.00 ± 1.39. Comparisons between the two groups showed no statistically significant differences in parameters other than gender (*p* > 0.05).

**TABLE 1:** Demographics and clinicopathologic characteristics of the training and validation sets.

Variables	Overall (*n* = 1373)	Training set (*n* = 962)	Validation set (*n* = 411)	*p*‐Value
Age (years)	59.75 ± 9.25	59.71 ± 9.30	59.83 ± 9.13	0.826
KPS	78.10 ± 9.39	77.94 ± 9.38	78.47 ± 9.39	0.338
Gender (%)				
Female	258 (18.79)	196 (20.37)	62 (15.09)	0.026
Male	1115 (81.21)	766 (79.63)	349 (84.91)	
BMI (kg/m^2^, %)				
18.5–23.9	768 (55.94)	543 (56.44)	225 (54.74)	0.883
24–27.9	405 (29.50)	278 (28.90)	127 (30.90)	
≥28	78 (5.68)	54 (5.61)	24 (5.84)	
<18.5	122 (8.89)	87 (9.04)	35 (8.52)	
TNM (%)				
III	236 (17.19)	157 (16.32)	79 (19.22)	0.220
IV	1137 (82.81)	805 (83.68)	332 (80.78)	
Surgery (%)				
No	774 (56.37)	539 (56.03)	235 (57.18)	0.739
Yes	599 (43.63)	423 (43.97)	176 (42.82)	
Radiation (%)				
No	810 (58.99)	572 (59.46)	238 (57.91)	0.634
Yes	563 (41.01)	390 (40.54)	173 (42.09)	
Chemotherapy (%)				
No	79 (5.75)	53 (5.51)	26 (6.33)	0.639
Yes	1294 (94.25)	909 (94.49)	385 (93.67)	
Targeted (%)				
No	823 (59.94)	576 (59.88)	247 (60.10)	0.987
Yes	550 (40.06)	386 (40.12)	164 (39.90)	
WBC (10^9^/L)	8.72 ± 4.52	8.70 ± 4.48	8.76 ± 4.63	0.801
Hb (g/L)	119.75 ± 17.28	119.99 ± 17.16	119.18 ± 17.57	0.427
PLT (10^9^/L)	230.00 ± 84.95	230.72 ± 84.13	228.30 ± 86.92	0.628
LDH (U/L)	232.16 ± 125.18	234.96 ± 133.16	225.59 ± 104.01	0.204
MAO (U/L)	6.72 ± 2.82	6.72 ± 2.83	6.74 ± 2.82	0.887
PT (S, %)	15.84 ± 2.76	15.80 ± 2.28	15.93 ± 3.64	0.443
APTT (S, %)	27.52 ± 2.58	27.50 ± 2.66	27.57 ± 2.39	0.610
Ddimer (mg/L)^a^	0.67 (0.37, 1.38)	0.67 (0.37, 1.34)	0.69 (0.38, 1.49)	0.282
FIB (g/L)	4.00 ± 1.39	3.96 ± 1.38	4.09 ± 1.41	0.108
FDP (μg/mL)^a^	2.50 (2.50, 4.10)	2.50 (2.50, 4.07)	2.50 (2.50, 4.05)	0.963
T (%)	920.67 ± 444.73	913.18 ± 445.21	938.20 ± 443.66	0.340
B (%)^a^	91.00 (53.00, 148.00)	90.00 (53.00, 147.00)	94.00 (51.00, 149.50)	0.396
NK (%)^a^	219.00 (143.00, 341.00)	219.00 (139.25, 328.50)	220.00 (152.00, 363.50)	0.204

^a^ Expressed as median (M) and interquartile range (IQR).

### 2.2 Factors influencing VTE in the training set

Table 2 displays the influencing factors of VTE in the training set. The results of univariate analysis have identified potential factors influencing VTE, including variables such as Age, KPS, TNM, and Surgery, among 13 others (*p* < 0.2). The results of the multivariate stepwise regression showed that Age, KPS, Chemotherapy, Targeted, WBC, PLT, LDH, MAO, D‐dimer and FIB are influencing factors for VTE. Specifically, the impacts are as follows: Age [OR 1.03 (95% CI: 1.00–1.06, *p* = 0.030)], KPS [OR 0.96 (95% CI: 0.94–0.99, *p* = 0.002)], Chemotherapy [OR 7.52 (95% CI: 1.05–59.77, *p* = 0.046)], Targeted [OR 2.25 (95% CI: 1.33–3.79, *p* = 0.002)], WBC [OR 1.06 (95% CI: 1.01–1.12, *p* = 0.022)], PLT [OR 1.01 (95% CI: 1.01–1.01, *p* = 0.026)], LDH [OR 1.01 (95% CI: 1.01–1.02, *p* = 0.004)], MAO [OR 1.10 (95% CI: 1.01–1.20, *p* = 0.024)], Ddimer [OR 1.24 (95% CI: 1.13–1.37, *p* < 0.001)], FIB [OR 1.22 (95% CI: 1.03–1.45, *p* = 0.025)].

**TABLE 2:** Logistic regression analysis of the risk factors for VTE in the training set.

Variables	Without VTE (*n* = 879)	VTE (*n* = 83)	OR (univariable)	OR (multivariable)
Age (years)	59.58 ± 9.35	61.13 ± 8.74	1.02 (0.99–1.04, *p* = 0.146)	1.03 (1.00–1.06, *p* = 0.030)
KPS	78.38 ± 9.32	73.25 ± 8.85	0.95 (0.93–0.97, *p* < 0.001)	0.96 (0.94–0.99, *p* = 0.002)
Gender (%)				
Female	175 (19.91)	21 (25.30)		
Male	704 (80.09)	62 (74.70)	0.73 (0.44–1.24, *p* = 0.245)	
BMI (kg/m^2^, %)				
18.5–23.9	492 (55.97)	51 (61.45)		
24–27.9	259 (29.47)	19 (22.89)	0.71 (0.41–1.22, *p* = 0.216)	
≥28	50 (5.69)	4 (4.82)	0.77 (0.27–2.22, *p* = 0.631)	
<18.5	78 (8.87)	9 (10.84)	1.11 (0.53–2.35, *p* = 0.779)	
TNM (%)				
III	149 (16.95)	8 (9.64)		
IV	730 (83.05)	75 (90.36)	1.91 (0.90–4.05, *p* = 0.090)	
Surgery (%)				
No	486 (55.29)	53 (63.86)		
Yes	393 (44.71)	30 (36.14)	0.70 (0.44–1.12, *p* = 0.135)	
Radiation (%)				
No	528 (60.07)	44 (53.01)		
Yes	351 (39.93)	39 (46.99)	1.33 (0.85–2.09, *p* = 0.212)	
Chemotherapy (%)				
No	52 (5.92)	1 (1.20)		
Yes	827 (94.08)	82 (98.80)	5.16 (0.70–37.78, *p* = 0.106)	7.52 (1.05–59.77, *p* = 0.046)
Targeted (%)				
No	541 (61.55)	35 (42.17)		
Yes	338 (38.45)	48 (57.83)	2.20 (1.39–3.46, *p* < 0.001)	2.25 (1.33–3.79, *p* = 0.002)
WBC (10^9^/L)	8.49 ± 4.36	10.85 ± 5.10	1.10 (1.06–1.15, *p* < 0.001)	1.06 (1.01–1.12, *p* = 0.022)
Hb (g/L)	120.14 ± 17.10	118.47 ± 17.80	0.99 (0.98–1.01, *p* = 0.398)	
PLT (10^9^/L)	227.63 ± 81.43	263.45 ± 103.81	1.01 (1.01–1.01, *p* < 0.001)	1.01 (1.01–1.01, *p* = 0.026)
LDH (U/L)	228.56 ± 110.37	302.76 ± 268.86	1.01 (1.01–1.01, *p* < 0.001)	1.01 (1.01–1.02, *p* = 0.004)
MAO (U/L)	6.61 ± 2.77	7.81 ± 3.19	1.15 (1.06–1.23, *p* < 0.001)	1.10 (1.01–1.20, *p* = 0.024)
PT (S, %)	15.80 ± 2.27	15.81 ± 2.46	1.00 (0.91–1.11, *p* = 0.962)	
APTT (S, %)	27.49 ± 2.63	27.59 ± 3.00	1.01 (0.93–1.10, *p* = 0.732)	
Ddimer (mg/L)^a^	0.62 (0.34, 1.24)	1.35 (0.69, 3.28)	1.26 (1.15–1.38, *p* < 0.001)	1.24 (1.13–1.37, *p* < 0.001)
FIB (g/L)	3.89 ± 1.33	4.70 ± 1.65	1.47 (1.26–1.71, *p* < 0.001)	1.22 (1.03–1.45, *p* = 0.025)
FDP (μg/mL)^a^	2.50 (2.50, 3.60)	12.10 (3.80, 12.50)	1.03 (1.00–1.06, *p* = 0.051)	
T (%)	914.06 ± 448.38	903.87 ± 412.49	1.00 (1.00–1.02, *p* = 0.842)	
B (%)^a^	88.00 (52.00, 149.00)	99.00 (64.50, 127.50)	1.00 (1.00–1.01, *p* = 0.561)	
NK (%)^a^	218.00 (140.00, 328.00)	225.00 (136.50, 324.50)	1.00 (1.00–1.01, *p* = 0.965)	

^a^ Expressed as median (M) and interquartile range (IQR).

### 2.3 Establishment and evaluation of visualized models

**FIGURE 2:**
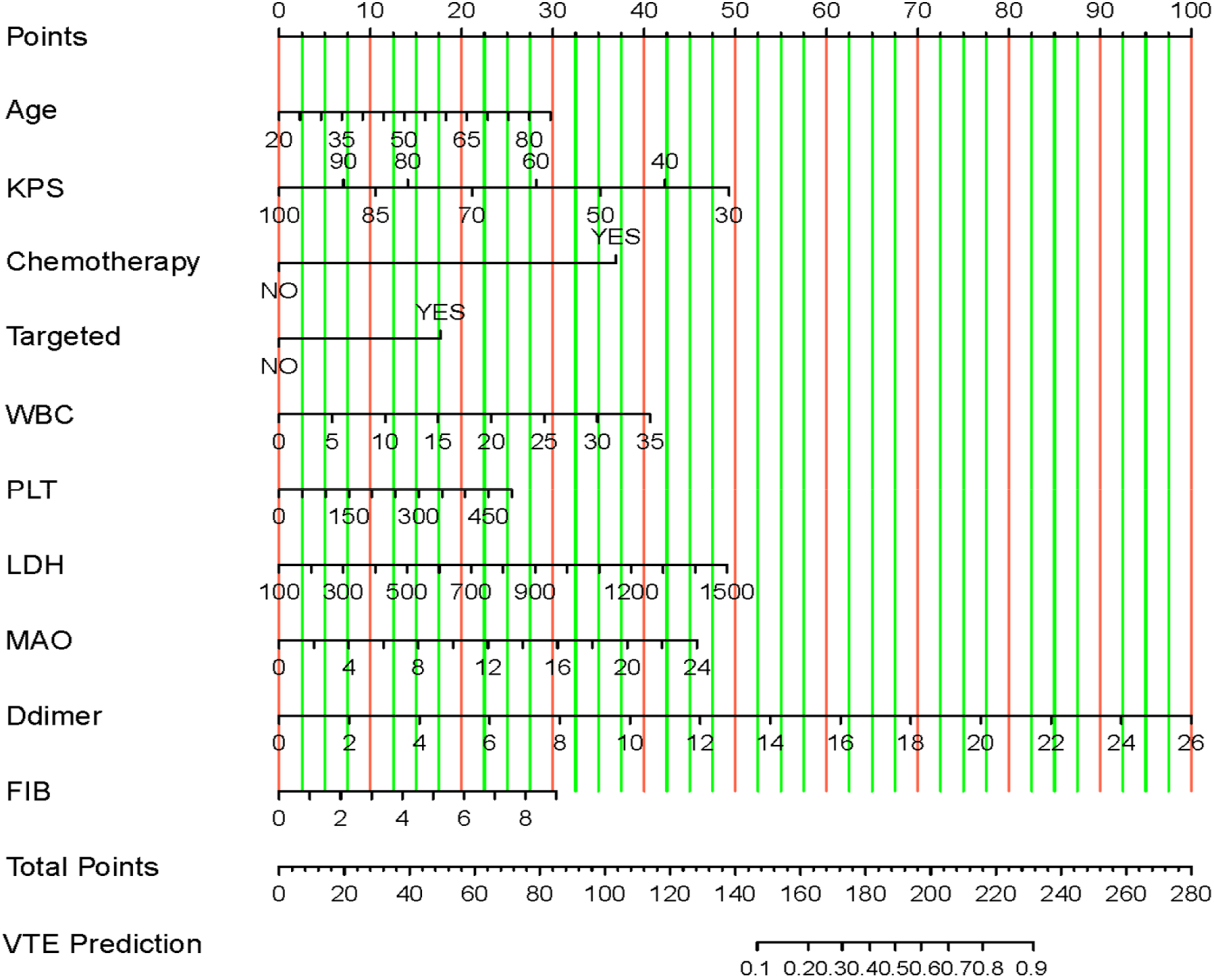
The nomogram model was constructed based on the independent risk factors.

A final predictive model was constructed using the features selected from the stepwise regression, including Age, KPS, Chemotherapy, Targeted therapy, WBC, PLT, LDH, MAO, D‐dimer, and FIB. A visual nomogram for this model is depicted in Figure 2.

**FIGURE 3:**
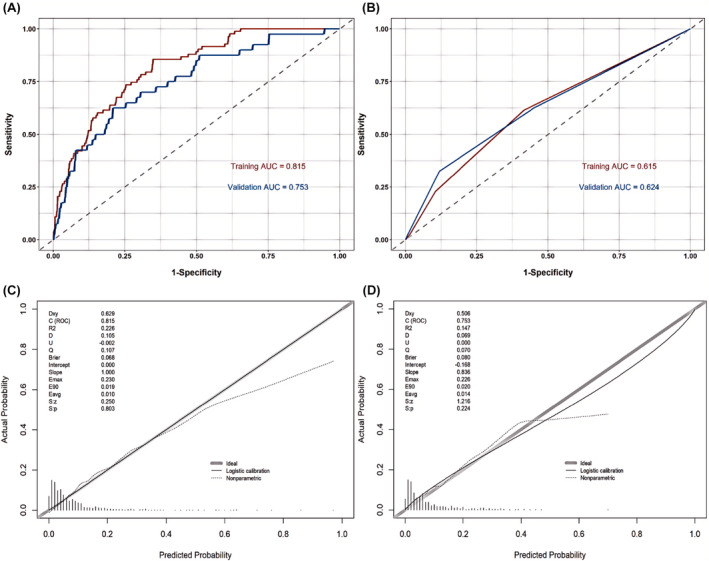
The predictive effectiveness of the nomogram. (A) ROC curves of the nomogram, (B) ROC curves of the Khorana, (C) calibration plot in the training set, (D) calibration plot in the validation set.

Additionally, the ROC curves for the model were plotted, with the area under the ROC curve (ROC‐AUC) for the training set is 0.815 (95% CI 0.772–0.858), and for the validation set, the ROC‐AUC was 0.753 (95% CI 0.672–0.835). These results demonstrate that the model has good discriminatory ability, effectively differentiating between groups (Figure 3A). For both the training and validation sets, the Brier scores were calculated to be 0.068 and 0.080, respectively, indicating reliable predictive performance as both scores are below the 0.25 threshold. Furthermore, the Hosmer and Lemeshow tests yielded *p*‐values of 0.154 and 0.292 in the training and validation sets, respectively, suggesting no significant lack of fit since both values are above 0.05. Additionally, a calibration curve in Figure 3C,D showed good agreement between predicted and observed outcomes, further affirming the model's effectiveness.

**FIGURE 4:**
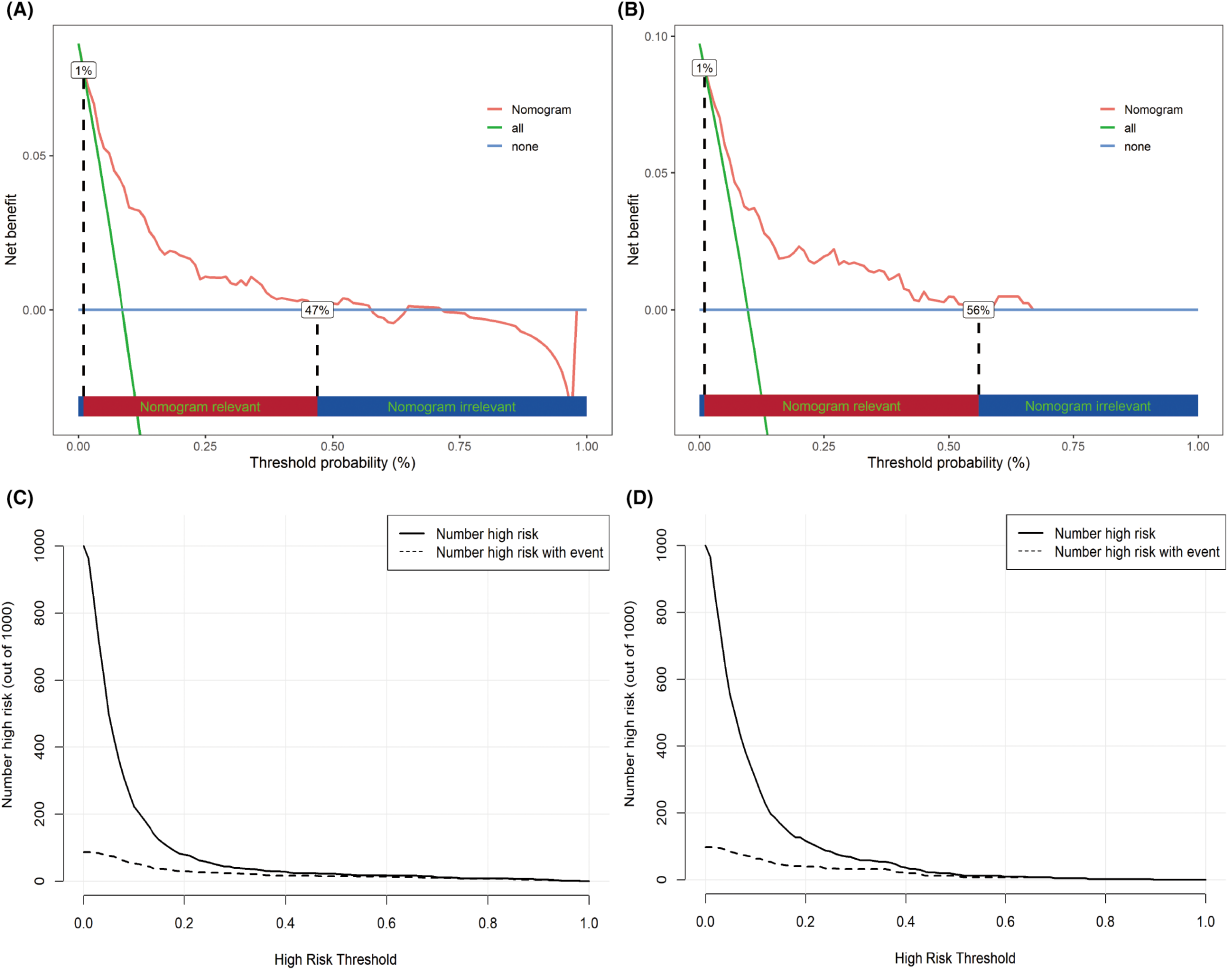
Decision curve analysis (DCA) and clinical impact curves (CIC) for the nomogram (A) DCA for the training set, (B) DCA for the validation set, (C) CIC for the training set, (D) CIC for the validation set.

The clinical utility of the model was evaluated through the CIC and DCA methods. These assessments revealed that the predictive model offers a substantial net benefit across the probability thresholds of 0.01–0.47 for the training set and 0.01–0.56 for the validation set (Figure 4).

### 2.4 Comparison of the nomogram model with Khorana

To further assess the predictive value and clinical utility of our model, we conducted a comparison with the classic Khorana for VTE. The Khorana exhibited a ROC‐AUC of 0.615 (95% CI 0.555–0.675) for the training set and 0.624 (95% CI 0.531–0.716) for the validation set (Figure 3B), both significantly lower than the ROC‐AUCs of our nomogram model. Table 3 reveals that, in comparison, the high‐risk group identified by our newly developed nomogram model displayed superior accuracy in both the training and validation sets than the Khorana, with percentages of 18.62% versus 17.12% and 24.51% versus 22.81%, respectively. Conversely, accuracy for the low‐risk group in both the training and validation sets was lower with our nomogram model than the Khorana, at 2.22% versus 7.52% and 4.85% versus 7.63%, respectively. This suggests that our nomogram model allows for a more precise risk segmentation.

**TABLE 3:** Comparison of accuracy between nomogram model and Khorana (%).

Data sets	VTE	Nomogram		Khorana	
		Low‐risk	High‐risk	Low‐risk	High‐risk
Training set	Not VTE	573 (97.78)	306 (81.38)	787 (92.48)	92 (82.88)
	VTE	13 (2.22)	70 (18.62)	64 (7.52)	19 (17.12)
Validation set	Not VTE	294 (95.15)	77 (75.49)	327 (92.37)	44 (77.19)
	VTE	15 (4.85)	25 (24.51)	27 (7.63)	13 (22.81)

## 3 DISCUSSION

As the global population ages, the impact of lung cancer continues to grow, representing a significant challenge to increasing life expectancy in China and around the world.^15^, ^16^ The advent of ICI therapy has been transformative in the lung cancer arena, offering patients extended survival. However, VTE is a notable side effect of ICIs, highlighting the need for meticulous attention. Despite the availability of current risk assessment models like the Caprini,^17^ Padua,^18^ and Khorana scores,^19^ their effectiveness and accuracy in evaluating VTE risk among cancer patients remain constrained. Specifically, the lack of a precise predictive model for VTE risk in lung cancer patients undergoing ICI treatment is evident.^20^, ^21^ Nomogram models, converting intricate regression equations into straightforward, user‐friendly tools, excel in predicting the likelihood of individual outcomes with precision.^22^, ^23^ These models have effectively gauged VTE risk across various cancers, including lung cancer,^14^ gynecologic tumors,^13^ and hematologic malignancies.^24^ Notably, a study established a nomogram for predicting VTE risk in lung cancer patients, incorporating variables like performance status, tumor staging, and factors indicative of treatment and physiological status, validating its accuracy internally.^14^

Building on this groundwork, our study introduces a novel predictive model to assess VTE risk among lung cancer patients treated with ICIs. This comprehensive model integrates a wide array of factors, encompassing patient demographics, cancer treatment history, and results from immunologic and coagulation tests, thereby offering a holistic view of the patient's health and treatment landscape. Such inclusivity enhances the model's representation and utility. Moreover, with immunotherapy becoming increasingly prevalent in China and the intricate mechanisms by which it may induce VTE not fully understood, there needs to be a palpable gap in clinical guidance and specific VTE prevention strategies related to ICI treatment. Our establishment of a nomogram model identifies critical risk factors for VTE and underscores its precision in risk prediction through robust internal validation. By doing so, we shed light on vital insights for VTE prevention in lung cancer treatment with ICIs, marking a step forward in patient care and safety.

Age, PLT, D‐dimer, and FIB are widely acknowledged as reliable indicators for VTE, supported by numerous studies^25^, ^26^, ^27^, ^28^ that have established a direct correlation with VTE. Within our predictive model for lung cancer patients receiving ICI treatment, these factors also emerge as significant predictors of VTE. This alignment with prior research underscores the accuracy of our nomogram model in reflecting the clinical indicators for VTE risk. Additionally, existing literature highlights an elevated VTE risk among cancer patients undergoing chemotherapy and targeted therapy.^29^ Our model corroborates these findings, identifying both treatment modalities as risk enhancers for VTE patients treated with ICIs. This heightened risk may stem from the compounded effects of ICIs with chemotherapy or targeted agents, exacerbating damage to vascular endothelial cells and altering the balance of procoagulant proteins and anticoagulants. LDH, a marker of tumor burden, disease activity, and tissue damage, has been implicated as a significant predictor of VTE risk prior to chemotherapy.^30^ Significant correlations between elevated LDH levels and increased VTE risk have been documented in testicular germ cell tumors and prostate cancer, with some studies indicating that LDH levels exceeding 198 U/L nearly triple the VTE risk.^31^, ^32^, ^33^ Consistently, our model identifies LDH as a critical risk factor for VTE in lung cancer patients post‐ICI treatment, in agreement with existing evidence. The role of MAO, a copper‐containing enzyme comprising MAO‐A and MAO‐B isoenzymes, in VTE formation is also explored. Notably, the reactive oxygen species (ROS) generated by MAO‐A can impair the contractility of vascular endothelial cells, leading to endothelial dysfunction, inflammation, and damage, which our predictive model suggests could contribute to VTE in our patient cohort.^34^, ^35^ MAO may have implications in predicting VTE due to its involvement in biological processes and disease pathways.

While there are currently no research papers directly addressing the relationship between MAO and VTE predictions, further studies exploring the association between MAO activity, genetic variations, and VTE risk factors could provide valuable insights into the predictive value of MAO in identifying individuals at risk of developing VTE. This proposed mechanism, while compelling, necessitates further investigation to be definitively confirmed. In addition, Mao's activity may vary widely among individuals. This variability makes it difficult to standardize measurements across populations. Elevated WBC counts have been linked with a higher incidence of VTE in cancer patients, possibly through the interaction between P‐selectin glycoprotein ligand 1 (PSGL‐1) on leukocytes and P‐selectin, promoting the release of procoagulant microparticles.^36^, ^37^, ^38^, ^39^ Our findings align with this hypothesis, underscoring WBCs as a significant risk factor for VTE. Lastly, an intriguing association is observed between the KPS score and VTE. Our study reveals a positive correlation, suggesting that higher KPS scores, indicative of better functional status and shorter periods of bed rest, are associated with reduced thrombus formation rates, thus reflecting real‐world clinical observations.

Thromboprophylaxis plays a pivotal role in lowering mortality rates among lung cancer patients. Carefully chosen predictive models have the potential to lighten the clinical workload significantly. Nomogram models, for instance, provide an accurate estimate of the VTE risk in lung cancer patients undergoing ICI therapy, pinpointing those at high risk who might benefit from targeted interventions. However, given the relatively recent adoption of ICIs, further research involving larger patient populations and more comprehensive data is imperative to solidify the link between ICIs and VTE.

The study's validity may be compromised by its reliance on retrospective data and single‐centre design, which heighten the risk of selection and reporting biases, potentially limiting the universality of the findings. This concern is particularly relevant given the study's narrow focus on a specific group of lung cancer patients in China. To mitigate these issues, it is crucial for future research to prospectively validate the nomogram model across a broader spectrum of patient populations. Additionally, the initial development of the model, based on only 22 features, might have overlooked critical factors fundamental to a thorough VTE risk assessment. Recognizing the importance of incorporating additional potential risk factors is essential, as is the commitment to the continuous refinement of the model. These strategies are crucial to enhancing the model's accuracy and, consequently, the efficacy of predicting VTE risk in lung cancer patients undergoing ICI therapy. Finally, this is a retrospective study with unavoidable limitations in study design, such as recall and recording biases. In the future, we should carry out prospective cohort studies to circumvent these limitations and make our models more accurate and universal. We are optimistic about future research endeavors to advance VTE prevention, especially those focused on evaluating VTE risk related to immunotherapy and developing tailored prevention and anticoagulation strategies.

## AUTHOR CONTRIBUTIONS

**Guanzhong Liang:** Conceptualization (lead); investigation (lead). **Zuhai Hu:** Formal analysis (equal); methodology (equal); resources (equal); software (equal). **Qianjie Xu:** Data curation (equal); formal analysis (equal). **Guixue Wang:** Project administration (equal); supervision (equal); writing – review and editing (equal). **Ying Wang:** Project administration (equal); supervision (equal). **Xiaosheng Li:** Data curation (equal); writing – original draft (equal); writing – review and editing (equal). **Wei Zhang:** Writing – review and editing (equal). **Haike Lei:** Data curation (lead); writing – review and editing (lead).

## FUNDING INFORMATION

This study was supported by grants from the Natural Science Foundation of Chongqing Municipality (CSTB2024NSCQ‐MSX0229) and Chongqing Science and Technology Bureau (CSTB2022TIAD‐GPX0066).

## CONFLICT OF INTEREST STATEMENT

The authors declare that they have no known competing financial interests or personal relationships that could have appeared to influence the work reported in this paper.

## ETHICS STATEMENT

In our research, we followed the Declaration of Helsinki's ethical principles concerning using human subjects in medical research. Chongqing University Cancer Hospital's Ethics Committee reviewed and approved research studies (Approval Number: CZLS2023343‐A). The patients/participants provided written informed consent to participate in this study.

## Data Availability

All supporting data can be obtained from the corresponding author upon reasonable request.
